# The Race to Replace PDE5i: Recent Advances and Interventions to Treat or Manage Erectile Dysfunction: Evidence from Patent Landscape (2016–2021)

**DOI:** 10.3390/jcm11113140

**Published:** 2022-05-31

**Authors:** Mohammed Monirul Islam, Nimbagal Raghavendra Naveen, Posina Anitha, Prakash S. Goudanavar, G. S. N. Koteswara Rao, Santosh Fattepur, Muhammad Muhitur Rahman, Predeepkumar Narayanappa Shiroorkar, Mohammed Habeebuddin, Girish Meravanige, Mallikarjun Telsang, Sreeharsha Nagaraja, Syed Mohammed Basheeruddin Asdaq, MD. Khalid Anwer

**Affiliations:** 1 Department of Biomedical Sciences, College of Clinical Pharmacy, King Faisal University, Al-Ahsa 31982, Saudi Arabia; 2 Department of Pharmaceutics, Sri Adichunchanagiri College of Pharmacy, Adichunchanagiri University, B.G. Nagar 571448, Karnataka, India; 3 Department of Pharmaceutics, Annamacharya College of Pharmacy, New Boyanapalli, Rajampet 516126, Andhra Pradesh, India; posina.anitha26@gmail.com (P.A.); pgoudanavar01@gmail.com (P.S.G.); 4 Department of Pharmacy, School of Medical and Allied Sciences, Galgotias University, Greater Noida 203201, Uttar Pradesh, India; drgsnkrao@gmail.com; 5 School of Pharmacy, Management and Science University, Seksyen 13, Shah Alam 40100, Selangor, Malaysia; 6 Department of Civil and Environmental Engineering, College of Engineering, King Faisal University, Al-Ahsa 31982, Saudi Arabia; mrahman@kfu.edu.sa; 7 Department of Biomedical Sciences, College of Medicine, King Faisal University, Al-Ahsa 31982, Saudi Arabia; pshiroorkar@kfu.edu.sa (P.N.S.); hmohammed@kfu.edu.sa (M.H.); gmeravanige@kfu.edu.sa (G.M.); 8 Department of Medicine, College of Medicine, King Faisal University, Al-Ahsa 31982, Saudi Arabia; mvtelsang@kfu.edu.sa; 9 Department of Pharmaceutical Sciences, College of Clinical Pharmacy, King Faisal University, Al-Hofuf, Al-Ahsa 31982, Saudi Arabia; sharsha@kfu.edu.sa; 10 Department of Pharmaceutics, Vidya Siri College of Pharmacy, Off Sarjapura Road, Bangalore 560035, Karnataka, India; 11 Department of Pharmacy Practice, College of Pharmacy, AlMaarefa University, Dariyah, Riyadh 13713, Saudi Arabia; sasdaq@gmail.com; 12 Department of Pharmaceutics, College of Pharmacy, Prince Sattam Bin Abdulaziz University, Al-Alkharj 11942, Saudi Arabia; m.anwer@psau.edu.sa

**Keywords:** erectile dysfunction, PDE5i, patent search, clinical trials

## Abstract

For a few decades, globally, erectile dysfunction (ED) has become more prominent even in young adults and represents a mounting health concern causing a significant effect on men’s quality of life. There is an expectation that by the end of 2025, the number of ED cases can rise to 322 million. We aimed to comprehensively analyze the scientific output of scholarly articles and studies in the field of ED (2016–2021). Data from scholarly articles were collected using Pubmed, and clinical trials-related information was accessed from the clinical trials website. An extensive patent search was conducted using databases such as USPTO (United States patent and trademark office) and EPO (European patent office), WIPO (World Intellectual Property Organization), etc. Owing to the high market value of ED drugs, considerable interest was attained to grab the opportunities. The race to replace the phosphodiesterase type 5 inhibitor (PDE5 inhibitor-PDE5i) can be identified as evident from the significant number of patents filed and the inventions cleared with clinical trials. Some other intriguing interventions are identified for ED treatment but have yet to gain public acceptance. The current analysis confirms the overall evolution and unexplored corners of research on ED treatment strategies with a current global projection.

## 1. Introduction

**Figure 1:**
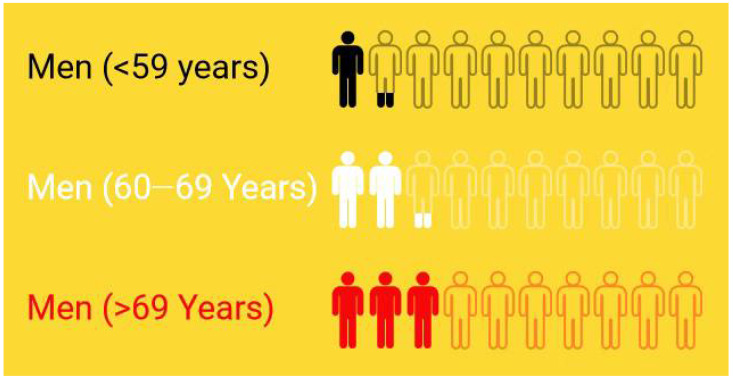
The ED prevalence distribution as per age of men.

**Figure 2:**
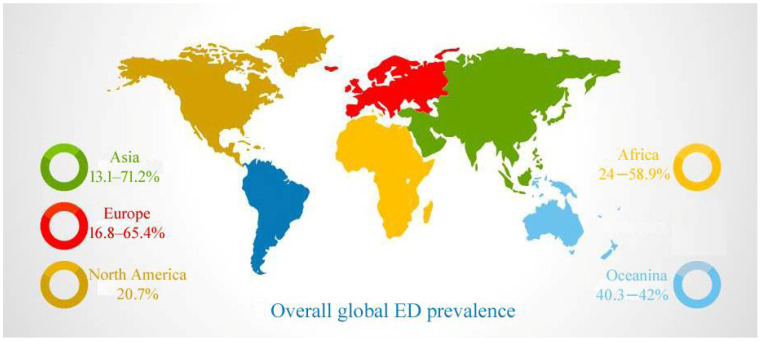
Overall global ED prevalence and the share by different continents.

Male sexual dysfunction envelops several disorders, including erectile dysfunction (ED), delayed or absent ejaculation, loss of sex drive, and premature ejaculation [1,2]. National Institutes of Health defined erectile dysfunction (ED) as a lack of ability to attain or maintain an erection sufficient to perform satisfactorily to complete the sexual activity. It causes severe distress and intensely affects personal relationships and overall confidence [3]. As per the five-item International Index of Erectile Function questionnaire [IIEF-5], ED severity has been classified based on scores obtained: severe with scores 1–7, 8–11 indicates moderate, 12–16 confirmed mild-moderate, and 17–21 and 22–25 indicates mild and no erectile dysfunction, respectively [4]. A total of 52% of men between the ages 40 and 70 years have shown some form of ED, according to the Massachusetts Male Aging Study (MMAS) [5]. The fact is that ED is a natural part of aging, and its prevalence rises with age. The prevalence of ED is 12% at age < 59 years, 22% in 60–69 years, and 30% in >69 years, as per a population-based study of U.S. health professionals [6] (Figure 1) (https://www.aafp.org/afp/2010/0201/p305.html, afp20100201p305-b3, accessed on 29 May 2022). Individuals with type 2 diabetes mellitus are at risk of ED three times higher than the general population [5,7] (https://www.aafp.org/afp/2010/0201/p305.html, afp20100201p305-b4, accessed on 29 May 2022). In another study, ED prevalence was found to increase with the age of individuals after 40 years. Around 9.1–49.9% and 54.9–94.7% of men of age less than 50 years and more than 70 years were suffering from at least one kind of ED. Figure 2 shows the global prevalence assessed as per IIEF-5, IIEF, or other IIEF variants in contrast to five continents. The overall global prevalence was identified as 13.1–71.2% [8]. Oceania occupies a significant share of this proportion [40.3–42%], followed by Africa with 24–58.9%. North America, Europe, and Asia stand in the consecutive positions.

ED frequency is most common in the US and southeastern and eastern Asian countries compared to Europe or South America. There is necessity to perform additional studies to recognize and differentiate actual genetic reasons from the impact of the environment on the occurrence of ED. From the data collected from Brazil and the Netherlands, it was clear that the prevalence rate of ED is significantly less (19–66 per thousand men) [9,10].

**Figure 3:**
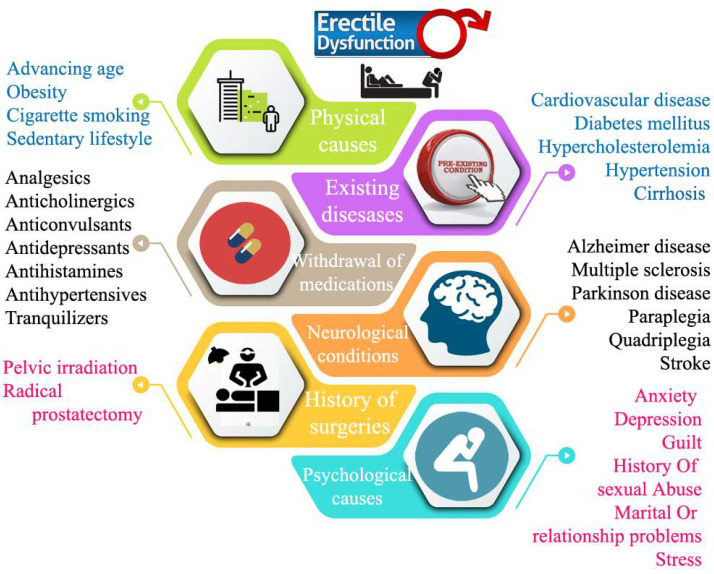
Risk factors associated with ED [13].

Reflex erection and psychogenic erection are two main facets of ED in which the former is under the control of lower parts of the spinal cord and peripheral nerves and it is accomplished by contact of the penile shaft, and the latter is under the limbic system of the brain and attained by erotic or emotional stimuli [4]. In general, the common erection is caused by parasympathetic and sympathetic neurons, which causes penile stimuli with sexual perception. By activating parasympathetic neurons, nitric oxide is produced from endothelial cells, which results in smooth muscle relaxation and the arterial influx of blood into the corpus cavernosum. The latter is followed by compression of the venous return, which produces an erection [11,12] (https://www.aafp.org/afp/2010/0201/p305.html, afp20100201p305-b6, accessed on 29 May 2022). Various risk factors associated with ED are shown in Figure 3. Advancing age, cardiovascular disease, smoking habits, diabetes mellitus, history of pelvic irradiation or surgery, etc. are impacting more for the occurrence of ED. Interestingly, withdrawal of medications can also contribute to or cause ED. Predominantly, special attention should be given to cardiovascular drugs such as lopid, digoxin, and gemfibrozil; antipsychotic drugs (pimazoide, haloperodiletc); and antidepressants. Clinical trials of oral ED medications, followed by withdrawal of offending drugs, prove to be unproductive in gaining sexual functionalities. This can aim the physician to an additional evaluation and alternative treatment.

There is no preferred initial diagnostic test for ED, and routine screening is not recommended. In most cases, a history and physical examination are enough to establish an accurate diagnosis of ED. Penile duplex ultrasound is not a helpful diagnostic test for ED [14]. ED is evaluated initially by total medical, sexual, and mental status stated by the American Urological Association (AUA) [15]. Approaches for ED therapy includes the first line: oral phosphodiesterase-5 inhibitors (PDE5i); second line: intracorporal and intraurethral injection therapy and vacuum constriction devices; and third line: penile prosthesis. Management options include lifestyle changes, oral drugs, penile injections, surgically implantable penile prostheses, and more innovative approaches such as externally tightened-up penile prostheses. Other emerging approaches such as shock wave therapy of the penis and injection of stem cells or platelet-rich plasma are still under consideration. These therapies have yielded promising initial outcomes and may be part of the ED treatment algorithm in the future.

The ED drugs market was valued at about USD 36.30 billion in 2020, and 56.53 million men are diagnosed with ED. There will always be considerable research on ED treatment due to its strong market potential. Until now, no one has focused on patents granted or applied for the treatment of ED and ongoing clinical trials information, with exceptional attention to Phase IV, as we intend to do. Several novel formulations, delivery systems, or methods and devices for ED management/treatment have been given special attention in the current discussion. Hopefully, in the coming days, all these recent advances can replace the PDE5i to treat/manage ED.

## 2. Clinical Trials Related to ED

### 2.1. Summary of Clinical Trials Data

To date, there is still no specific treatment/therapy for either complete treatment of ED. Hence, the researchers are always in line to invent new drug moieties or technologies. As per the need, around 506 interventions are in different phases of clinical trials (420-Interventional and 86-Observational). Sixty-two studies were in Phase I studies, while Phase 2 consisted of 71 interventions. At the same time, the rest 96 and 94 interventions were in Phase 3 and Phase 4 studies, respectively. Almost 45 interventions were completed, and others are in the recruiting stage. Surprisingly, 52 studies were performed by including female participants. Almost half of the clinical trials were sponsored by industries, and universities, organizations, and individuals shared the rest. The current section discusses the new process, devices, methods, and treatment strategies of ED unique to male sexual dysfunctions and pertinent to clinical trials (Phase IV) of candidate treatments in men. A detailed description of completed clinical trials is portrayed in Table 1 [16].

### 2.2. Clinical Trials on Tadalafil

“A Study in Patients With ED” investigated the efficiency of the long-acting drug tadalafil taken once a day or taken as needed, which results in longer treatment adherence and better long-term outcomes (over 24 weeks), compared with the short-acting drug sildenafil citrate. This study included 770 male participants 18 years and older (Adult, Older, Adult) with the inclusion criteria of a history of ED of at least three months duration and anticipated having the same adult female sexual partner during the study [17,18].

Eli Lilly and Company did a study using a multicenter, randomized, double-blind, placebo-controlled, fixed-dose, parallel design study to evaluate the effects on semen characteristics after 40 weeks of daily dosing with 20 mg tadalafil in 282 male participants 45 years and older with mild erectile dysfunction and specified semen characteristics [19].

In a study conducted by Bayer, phase IV clinical trials are ongoing to evaluate the efficacy of 10 mg vardenafil versus 10 mg tadalafil in the first 45 min post-dosing in an as-required regimen for four weeks in subjects with mild/moderate to severe ED. For this, they selected 759 heterosexual male participants with an age limit of 18–64 years with an ED of 6 months [20].

### 2.3. Clinical Trials on Vardenafil

Another study obtained information on the efficacy, safety, and duration of erection of the flexible-dose regime of vardenafil compared to placebo in patients with co-morbid factors associated with erectile dysfunction. The patients evaluated the duration of the end of the erection using an hourly chronometer during each attempt to have sex. High levels of total cholesterol or low HDL levels may cause atherosclerosis and lead to erectile dysfunction [21,22].

Vardenafil (Levitra) is registered with the Food and Drug Administration to treat erectile dysfunction. Although it is not intended to assess the effect of vardenafil on blood lipids, the study provides future-oriented data on men diagnosed with ED and dyslipidemia. The inclusion criteria for the survey were 395 heterosexual male participants with ED for more than six months with dyslipidemia, treated with a stable dosage of a statin, and an International Index of Erectile Function—Erectile Function domain score of <25, denoting mild to severe ED at Visit 2 [23,24].

A study titled “Evaluation of the Safety and Efficacy of vardenafil in Subjects with ED” investigated the safety, tolerability, and efficacy of vardenafil following a flexible-dose treatment schedule in subjects with varying etiology in selected 333 male participants. The study’s inclusion criteria were men with greater or equal to 18 with ED for 6 months and having a stable sexual relationship for more than six months. Another study was conducted on the effectiveness and tolerability of vardenafil in male patients with erectile dysfunction compared to a placebo. The study was done on 160 male participants 18 to 64 years old. To evaluate the influence of vardenafil on the self-esteem and self-confidence of subjects suffering from erectile dysfunction following treatment with vardenafil as compared to the placebo, at least four attempts to have sex (depending on the question in the subject’s diary) were made on four separate days during the 4-week unprocessed reporting period. They considered that at least 50 percent of attempts during this period must have been unsuccessful [25]. In the “Assessment of Duration of Erection with Vardenafil 10 mg”, they assessed the effect of 10 mg of vardenafil, taken on-demand in an ‘at home’ setting, compared to a placebo (dummy medication with no pharmacological activity). They considered the duration of erection withdrawn from the partner’s vagina as fruitful completion. The patient used the stopwatch for the above assessments. Questionnaires and patient diaries measured the efficacy of oral ED treatments. They selected 202 male participants aged 18–64 years with erectile dysfunction and stable heterosexual relationships [26].

### 2.4. Clinical Trials on Avanafil

In a study, they examined the therapeutic effects of two doses of avanafil after dosing in men with erectile dysfunction in 440 male monogamous heterosexual participants with six months of ED with the written agreement of at least four attempts at intercourse per month [27]. A survey entitled “Effect of Tadalafil on the Quality of Life and Sexual Life in Erectile Dysfunction” studied clinically and assessed the outcome of oral tadalafil 10 mg or 20 mg on psychosocial aspects and quality of life of erectile dysfunction patients and compared tadalafil with previous oral erectile dysfunction medicine. The duration of the study was 13 weeks. The study patients filled in a quality of life questionnaire and treatment satisfaction questionnaire. The study participants were 220 males 18 years or older with a history of erectile dysfunction (defined as a consistent alteration in the quality of erection that adversely affects the patient’s satisfaction with sexual congress) of at least three months duration that had been using any oral prescription medication, but not tadalafil, for erectile dysfunction for a minimum of 3 months before the visit.

## 3. Prospection of Scholarly Articles

**Figure 4:**
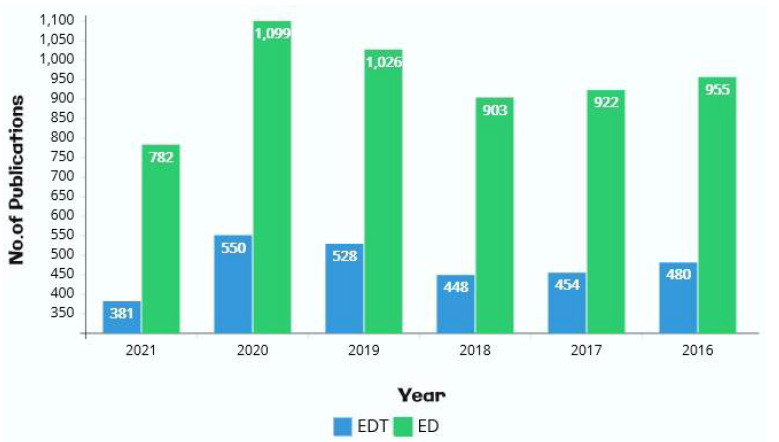
Comparison of No. of manuscripts published from 2016 to 2020 focusing on EDT and ED.

PubMed database was used for complete scientific analysis from 2016 to 2021 using two search queries: (a) Erectile dysfunction [EDT] [Title/Abstract] and treatment [Title/Abstract]) and (b) Erectile dysfunction [ED] [Title/Abstract], and the obtained results are represented in Figure 4. A total of 4334 manuscripts were published on ED; amongst them, 2142 were focused on treatment strategies.

A slight decline phase was identified for two years (2016–2018) with ED and EDT-related scholarly articles. However, from 2018 to 2020, a steady increase in the publications was identified, thus confirming the growing interest in the management of ED. The same scenario was observed even with EDT publications. A rapid rise was observed in the current year (2021) with the same phase. Until the 10th of August, 782 and 381 manuscripts were published on ED and EDT, respectively. This extensive research covered various recent advances in EDT, comparison of different treatment strategies of ED, applications of natural products, and lipid-based nanoparticles.

Lifestyle modification is always considered the first line of therapy, as is true in several medical circumstances. David F Mobley et al. reviewed the productive effect of this in the management of ED and overall health care. Despite the benefits, men expect the physician to help measure the immediate impact. Other advances in the management of ED such as vacuum constriction devices, intracavernosal injection, surgical treatment of penile prosthesis, and prostaglandin suppositories were also elaborated to upbring the knowledge for various researchers [28]. In 2004, the stem cell study for the ED treatment was carried out using embryonic stem cells. Stem cells accelerate the angiogenesis process with a consequent rise in cavernosal smooth muscles. To date, a total of 36 studies have been published on the basis of this concept; among them, two were in clinical trials [29]. The treatment of ED with tadalafil and sildenafil was compared in 16 trials and included a meta-analysis. The results confirmed that both had shown similar efficacies and adverse event rates. However, tadalafil improved the psychological outcomes very effectively, and thus was preferred by both patients and their partners.

Furthermore, lower flushing rate, higher back pain rates, and myalgia were observed with tadalafil in contrast to sildenafil [30]. Valerie Jia-En Sin et al. performed comprehensive literature on natural products and botanical medicine applications for EDT. While these products can serve as an impending source of several lead compounds, further studies are necessary to evaluate their safety and efficacy [31]. Very limited scientific information is available on the application of platelet-rich plasma injections for autologous cell therapy. Regardless of active marketing and public interest in these kinds of regenerative medicine, inadequate data is available on its clinical safety and potential adverse effects in EDF [32]. Another interesting approach in treating ED was low-intensity shockwave therapy. According to the study conducted by J C Angulo et al., this therapy has proven effective in short and medium terms. Nevertheless, the data on long-term efficiency is insufficient to conclude. However, more studies are needed to be done on the application of low-intensity shockwave therapy according to the different causes of ED [33]. In another study, sublingual apomorphine was studied for its effectiveness in patients who are unable to take PDE-5 inhibitors. Virginia Guillen and Jose Ra performed a systematic review and meta-analysis by comparing placebo. Available evidence proved that sublingual apomorphine is more effective with a dose of 2–6 mg and a treatment period of 4–8 weeks. Sublingual apomorphine is the only approved oral drug for ED, not enormously contraindicated with nitrates application [34].

Few reports stated that endovascular procedures of arteries related to erection are found to be safe in the selected group, but its benefits should be optimally studied for long-term follow-up [35]. Penile vascular surgery can be a feasible approach for the treatment of ED and found its position in getting natural spontaneous erection [36]. This surgery can be helpful, especially for patients suffering from corporaveno occulsive dysfunction. Integration ED drugs with various nanomaterials are of much interest in revolutionizing the clinical approach in the future. Lipid-based nanosystems possess superior therapeutic potential by improving their effectiveness and reducing possible side effects and drug susceptibility [37]. Several ED drugs were formulated into lipid-based systems, as depicted in Table 2.

## 4. Patent Analysis

**Figure 5:**
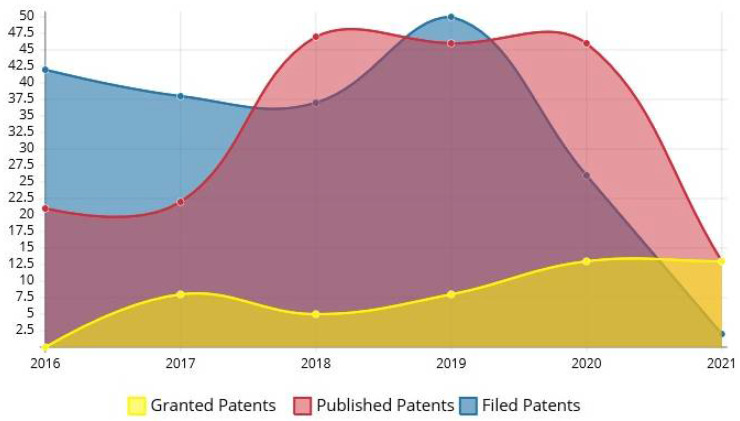
Patent documents by Published, Filed and Granted Date from 2016 to 2021.

The patent-related search was continued on ED after retrieval of publications data. Various patent databases such as USPTO (United States patent and trademark office) and EPO (European patent office), WIPO (World Intellectual Property Organization), etc. were used to extract the patent literature. Databases were searched with Title: (erectile AND dysfunction) AND Abstract: (erectile AND dysfunction) using the application filing date as a filter (1 January 2016 to 15 August 2021). A total of 225 patent documents were filed with various jurisdictions. The total number of patents filed, published, and granted from 2016 to 2021 is represented in Figure 5, and a list of granted patents is depicted in Table 3. The number of patents filed followed a zigzag pattern, but a surprisingly enormous increase in the published patents was observed. A total of 13 patents were granted in the first half of 2021, and still this number can be increased by the end of the year.

**Figure 6:**
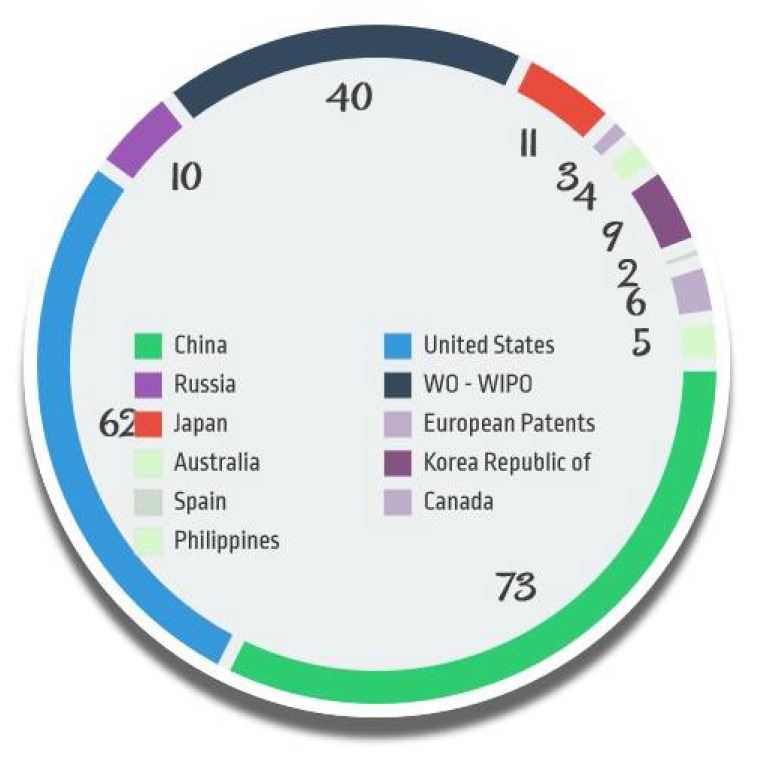
Patent documents on ED by Jurisdiction.

Jurisdiction wide sharing of the patents are shown in Figure 6. China tops the list with 73 documents followed by the United States with 62. A total of 40 patents were filed under WIPO and few patents were filed from various jurisdictions such as the Korea Republic of, Japan, Russia, etc.

### Patents Classification and Concordance

**Figure 7:**
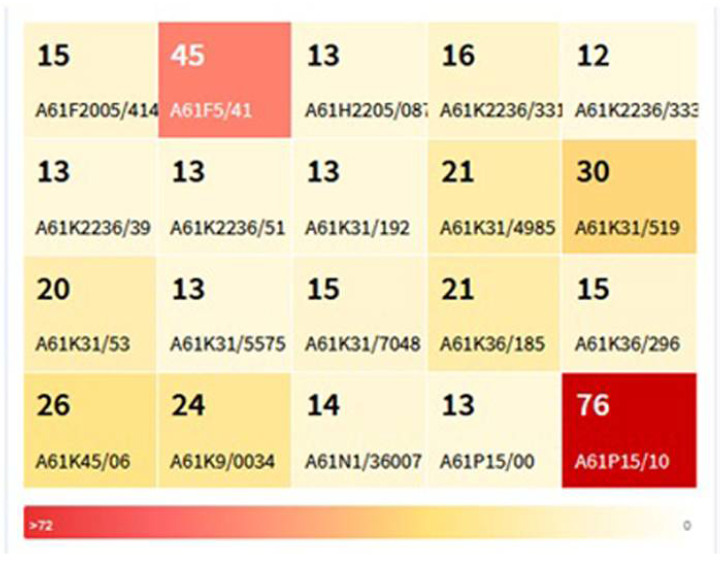
Distribution of ED patents as per CPC and IPC.

Patents’ classification helps in grouping filed patents as per their technical characteristics. This also aids most finely and accurately of patent search. There are several classifications used by many jurisdictions, owing to their patent laws. To consolidate the patents, all jurisdictions came forward to form standard technical codes. These are classified as Cooperative patent classification (CPC) and International patent classification (IPC). As an expansion to IPC, CPC came into the picture from Q4 of 2010. CPC was wholly managed by USPTO and EPO [49]. ED patents were classified, and the schematic distribution is represented in Figure 7. A61P15/10 class containing the inventions related to the specific therapeutic activity of chemical compounds or medicinal preparations for treating impotence has the highest number of patents (76). This was followed by A61F5/41, a particular class dedicated to implantable into blood vessels, prosthetic devices such as stents, contraceptive devices, dental prosthetics, and dressings or absorbents. Devices or methods adapted to bring pharmaceutical products into administering forms related inventions were grouped under the A61K class. A61H class contains devices for stimulating or locating reflex points in the body for electrotherapy, radiation therapy, ultrasound therapy, magnetotherapy, etc. Few inventions to promote penis erection or penis implants were grouped under A61F5/41. The scientific procedure of genitals massage is also included in the same class.

**Figure 8:**
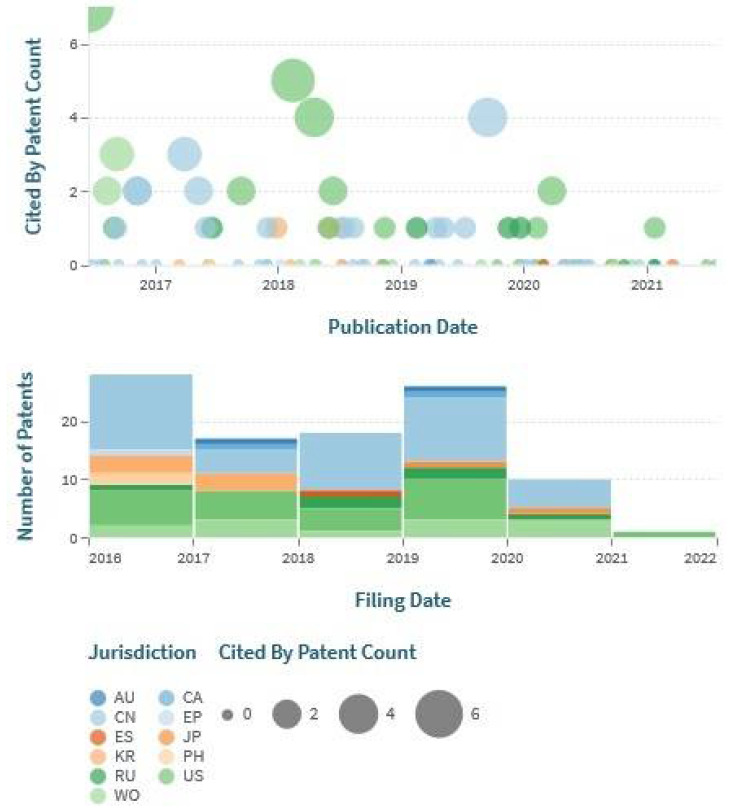
Graphical representation of ED patent citations concerning filing date and jurisdiction.

Citation frequency was further considered to measure the patent appropriateness. Figure 8 represents the filing date and number of patents with citations concerning different jurisdictions. Cited patent counts were higher for the patents published in 2018–2019, and the relevance was found to be more than 4. Few patents published with USPTO were shown the maximum number of citations through these given periods.

## 5. Technical Aspects of Few Patents on ED

Since 2006, numerous processes, devices, methods, and treatment strategies have received intellectual protection through patenting. To date, there is no literature comprehension of the present discussion intended. Many of the patents were granted for the invention of several methods for treating ED and thereof. US patent 20180064937A1 describes the various methods and devices for treating ED, which include reducing the blood outflow from penile tissue by delivering energy to it to cause remodeling of that specific tissue. Radiofrequency energy can be applied by configuring a few devices to thereby elevate the internal penile tissue. This predetermined temperature can initiate collagen fibers’ synthesis and/or regeneration to initiate angiogenesis and neovascularization by increasing oxygenation to endothelial cells. In another invention, a novel pharmaceutical composition of LDD175, co-administered with PDE5 inhibitors, was used for treating or preventing ED. LDD175 has significantly improved erectile functioning through superior corporal smooth muscle relaxation effect. The effectiveness of LDD175 was found to have an equivalent therapeutic effect as that of udenafil, a PDE5 inhibitor, and showed a synergistic effect with co-administration.

**Figure 9:**
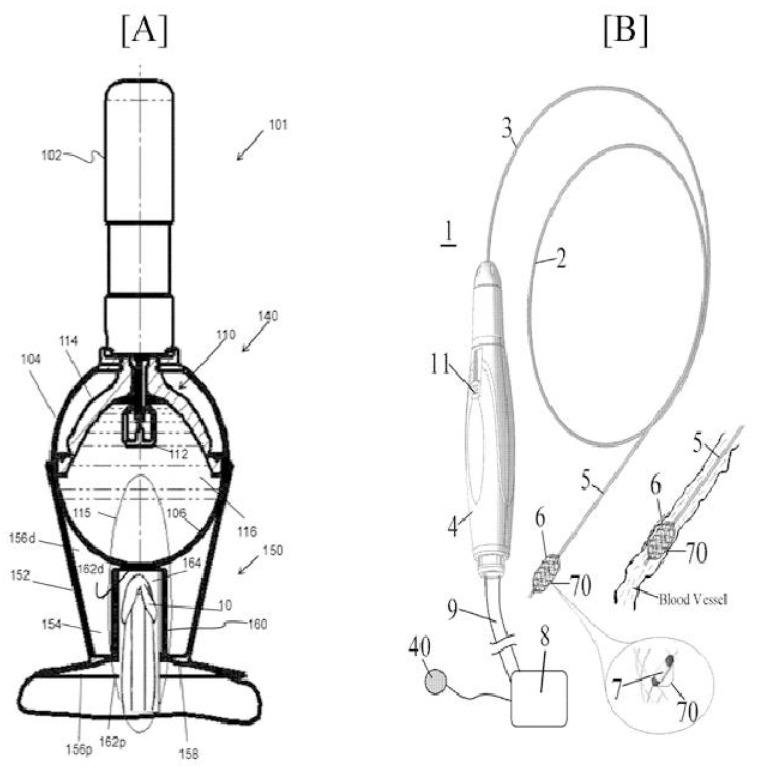
Schematic representation of (**A**) Illustrative shockwave producing device [US 2018/0228638-A1] (**B**) Catheter system [US 2020/0008871-A1].

A device producing extracorporeal shockwaves for the treatment of ED gained importance. Within this invention, the shockwave producing device comprises a controller and/or computer applications that control the shockwave treatment produced by the applicator (Figure 9A). This therapy can promote at least one or more therapies for treating ED, but is not limited to vasodilation, endothelial function, neuronal regeneration, reducing cytoplasmic calcium concentration, enzymatic inhibition, PDE5 inhibitors, etc. GaijunTeng et al. invented a method of treating or alleviating ED. This consists of multiple electrodes, placed within a segment of the internal iliac artery of the patient and against the blood vessel wall thereof, followed by application of radiofrequency energy of 60–180 for 120 s, which increases the temperature of nearby tissues (Figure 9B). This thermal alteration produces a lesion with 5–8 mm or 5.9–6.9 mm depth. An external unit can be coupled to a catheter to provide RF energy and temperature monitoring.

**Figure 10:**
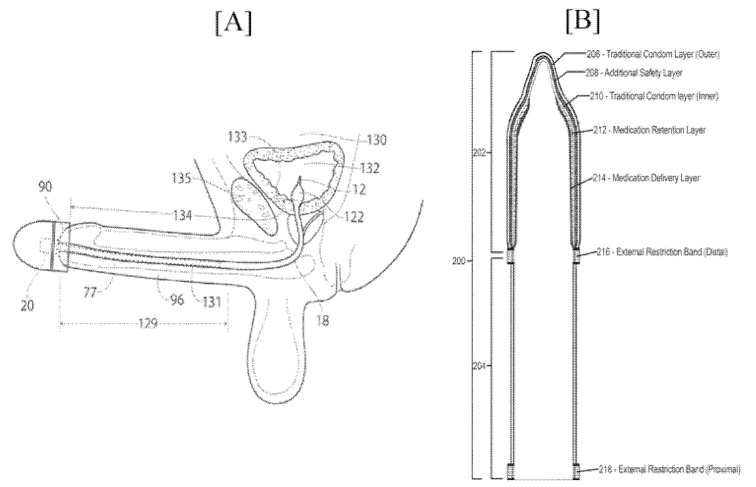
(**A**) Operation of ED device of invention US/2021/0161699-A1 and (**B**) Embodiment of a multi-layer male condom of the invention of US 10653549 B2.

BMR Medical LLC was granted a patent for three step treatment of ED. Starting with a predetermined course of external counter-pulsation, the treatment is applied to the patient’s lower body, followed by the low-intensity shock wave and finally carboxytherapy for a predetermined period. Another insertion assembly in communication with a distal end cap device, placed at a distal end of the ED device, and external to the user’s body provides a mechanical and electronic characteristic. The proximal end of this insertion is closed and provides a lumen of the wall of reduced thickness, providing local expansion of the lumen wall to an elastic bulb when fluid is inserted in the lumen (Figure 10A).

Carl W Lang, IV proposed a simple novel method of applying topical ED medicaments through a condom or like the prophylactic article for controlled release was granted with the patent in 2020. Additionally, this invention is the combination of initial ingestion of a small oral dose of ED medicaments. The initial oral application metabolizes and runs its course, while the topical application can provide the needed dose. Once the sexual activity is completed, the wearer can simply remove the topical system to return to the flaccid state as quickly as possible. This can assure safe implementation and reduce the propensity to damage the excessive erection states or priapism (Figure 10B).

Implantable penile prosthetic in EDT containing a pump with bulb includes the mid-section having a first face that is planar in longitudinal cross-section, a second concave face, and arced longitudinal cross-section. An inflatable cylinder will be implanted into the corpora cavernosum of the penis, and the reservoir will implant in the abdomen that communicates with the cylinder and pump. The squeezing of the bulb moves the liquid from the reservoir into the cylinders to provide the erection of the penis. However, users can feel the difficulty in grasping the pump bulb or in repeatedly squeezing the bulb during sexual activity. US Patent 10,940,033 B2 describes an exemplary method in treating ED by generating stimulation sessions with an electro-acupuncture device, implanted beneath the skin with a duty cycle of fewer than 0.05 secs. The stimulations will reach to target tissue location through the electrode array at an acupoint.

Different compositions of embodiment ginger and an amino acid, preferably L-arginine and/or L-citrulline, were used to treat age-related ED. The compositions contain natural ingredients such as *Paulliniacupana*, *Muirapuamacortex*, *Magnoliaeofficinalis*, and/or *Fructusaurantilimmaturus*. All these ingredients are administered in an adequate amount to treat ED. WO 2019/102501 A1 describes the invention that concerns a device for overcoming ED. This device comprises an annular band adapted to be applied between the root of the scrotum and the base of a penis. A capsule containing a supporting shaft to cooperate natural erection of the penis allows a consequent performance of intercourse that is functional and satisfactory for both subjects. Pivipi Labs PteLTD is granted a patent for representing 9-phenyl-Simm-octahydoselenoxantene for EDT in 5–20%. The foresaid molecule is formulated in tablets or capsules, reducing side effects by accompanying somatic pathologies. An exciting invention was made to diagnose the ED symptoms by Andreev Yurji et al. This portable consists of a module with a processor connected to an inductive pressure measurement sensor. A low-frequency generator is connected to a watchdog timer. The microprocessor measures the inductive value of pressure and records the value in the memory.

**Figure 11:**
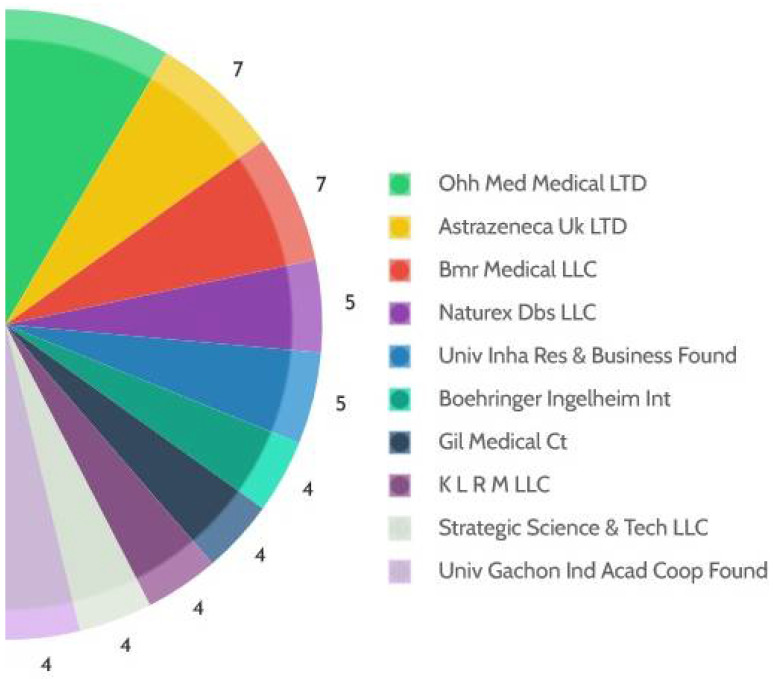
Top applicants and the number of patents filed in the area of ED since 2016.

Corpus cavernosum of penis-derived exosomes (CE) compositions were used along with a quasi-drug composition in treating ED efficiently. Distinct from conventional ED drugs, which generally attempt to improve the erection through an ephemeral rise in blood flow, CE increases the expression of different platelet endothelial cells, thus improving the tube formation ability. A fascinating invention was done by Biodlogics LLC in ED treatment using human berth materials such as placental and umbilical cord components, etc. A combination was made using one or more components of a placental organ selected: the umbilical cord, umbilical cord blood, chorionic membrane, amniotic fluid, placental globe, extracellular material, and others placental gelatin cells, etc. All these were homogenized with a tissue suspension solution [25% human albumin and dimethyl sulfoxide] and can be applied topically or introduced into a penis for the natural erection of the penis. Many of the researcher groups are continuously working to treat ED very effectively with reduced side effects and long-term benefits. As per the patents data obtained on ED, OHH Med Medical LTD have nine patent application, followed by AstraZeneca UK Ltd. and BMR Medical LLC, with seven patents filed. Other applicants such as Nature Dbs LLC, UnivInha Res & Business Found given their remarkable efforts to invent various methods/devices to treat ED (Figure 11)

## 6. Conclusions

To summarize the current manuscript, we have reviewed the different drugs/compositions, scholarly articles, and patent data for EDT. Treatments under various phases of clinical trials were elaborated with a particular focus on Phase-4. Owing to the high market value of ED drugs (36.25 billion USD in 2020), considerable interest was attained to grab the opportunities. Several researcher groups and industries have made great efforts in inventing various technologies/therapies to treat ED. This can be evident from the significant number of patents filed from 2016 to date. Many inventions were filed on methods/devices to improve the erection naturally. Many of these inventions were granted, as they were proven as efficient models in treating ED. Some intriguing interventions have identified the application of human birth material, corpus cavernosum of the penis, and other natural ingredients in treating ED, focusing on reducing the side effects and improving patient satisfaction. Despite this, many of these are still unable to gain public acceptance because of many unanswered issues. Hence, there will always be substantial scope to work on ED-related research/inventions.

## Figures and Tables

**Table 1:** List of completed clinical trials in ED [16].

S. No.	Study Title	Interventions	Locations
1.	A Study in Patients With Erectile Dysfunction	Drug: Tadalafil Drug: Sildenafil Citrate	Carpentras, France Chambery, France La Bouexiere, France
2.	Vardenafil and Cognitive-behavioral Sex Therapy for the Treatment of Erectile Dysfunction (STEDOV)	Drug: Vardenafil Behavioral: CBST	AmbulatoriMedicinadellaSessualità e Andrologia Florence, Italy
3.	Evaluation of the Safety and Efficacy of Vardenafil in Subjects With Erectile Dysfunction	Drug: Levitra (Vardenafil, BAY38-9456)	
4.	Assessment of Vardenafil in Patients With Erectile Dysfunction in Asia	Drug: Vardenafil (Levitra, BAY38-9456) Drug: Placebo	Hong Kong, Jakarta, Indonesia Petlaing Jaya, Salangor, Malaysia
5.	Evaluating of the Efficacy and Safety of Vardenafil in Subjects With Erectile Dysfunction	Drug: Levitra (Vardenafil, BAY38-9456)	
6.	Evaluating the Efficacy Vardenafil 10 mg vsTadalafil 10 mg in in Subjects With Erectile Dysfunction (ED)	Drug: Levitra (Vardenafil, BAY38-9456) Drug: Tadalafil	
7.	Investigate the Responsiveness of the Erectile Quality Scale to Vardenafil Flexible Dose vs. Placebo in Males With Erectile Dysfunction (ED)	Drug: Placebo Drug: Levitra (Vardenafil, BAY38-9456)	Phoenix, Arizona, United States Beverly Hills, California, United States Laguna Hills, California, United States
8.	Efficacy and Safety of Lodenafil Carbonate in the Treatment of Erectile Dysfunction in Patients With Diabetes.	Drug: lodenafil carbonate	SP, Brazil
9.	BAY38-9456, 5/10/20 mg, vs.Placebo in Erectile Dysfunction	Drug: Levitra (Vardenafil, BAY38-9456) Drug: Placebo	Adana, Turkey
10.	Evaluation of Efficacy and Safety of Vardenafil in Patients With Erectile Dysfunction and Type 1 Diabetes	Drug: Levitra (Vardenafil, BAY38-9456) Drug: Placebo	
11.	The Efficacy and Safety of New Herbal Formula (KBMSI-2) in the Treatment of Erectile Dysfunction	Drug: KBMSI-2	Department of Urology, Pusan National University Hospital Busan, Korea,
12.	Assessing Efficacy and Safety of Flexible Dosing With Vardenafil in Subjects With Erectile Dysfunction and Hyperlipidemia	Drug: Levitra (Vardenafil, BAY38-9456) Drug: Placebo	
13.	A Study of Tadalafil (LY450190) in Chinese Men With Erectile Dysfunction	Drug: Tadalafil	Beijing, China Changsha, China
14.	Research Evaluating a PDE5 Inhibitor for Erectile Dysfunction	Drug: Placebo Drug: Avanafil 100 mg Drug: Avanafil 200 mg	Jupiter, Florida, United States Raleigh, North Carolina, United States Wilmington, North Carolina, United States
15.	Satisfaction, Confidence and Naturalness in Men With Psychogenic Erectile Dysfunction (ED)	Behavioral: Group Psychotherapy Other: Group Psychotherapy plus Sildenafil citrate Drug: Sildenafil citrate	
16.	Evaluation of LEVITRA to Advance the Treatment of Erectile Dysfunction	Behavioral: Levitra (Vardenafil, BAY38-9456) Other: No Education	
17.	A Study Evaluating Vardenafil Compared to Placebo in Subjects With Erectile Dysfunction (ED) and Dyslipidemia	Drug: LEVITRA (vardenafil) Drug: placebo	Investigational Site Fairhope, Alabama, United States Investigational Site Homewood, Alabama, United States Investigational Site Huntsville, Alabama, United States
18.	A Study of Tadalafil and Sildenafil in Men With Erectile Dysfunction in China	Drug: Tadalafil Drug: Sildenafil	Beijing, China Changsha, China Hangzhou, China
19.	L-Arginine and Erectile Dysfunction	Drug: L-arginine Drug: Placebo	Institute of Clinical Pharmacology, Hannover Medical School Hannover, Lower Saxony, Germany
20.	Effect of Tadalafil on the Quality of Life and Sexual Life in Erectile Dysfunction	Drug: tadalafil	Tampere, Finland
21.	Daily Avanafil for Erectile Dysfunction	Drug: Avanafil 50 MG Drug: Placebo oral tablet	Faculty of Medicine, Alexandria University. Alexandria, Elazareta, Egypt
22.	A Randomized, Open-Label, Crossover, Multicenter, Single Dose Comparator Study Evaluating Onset Of Penile Rigidity In Men With Erectile Dysfunction Who Are Treated With Sildenafil And Tadalafil	Drug: tadalafil Drug: sildenafil	Pfizer Investigational Site Oslo, Norway Leeds, UK
23.	A Study in Erectile Dysfunction	Drug: Placebo Drug: Tadalafil	Indianapolis, Indiana, United States
24.	Evaluating the Efficacy of Vardenafil in Subjects With Erectile Dysfunction (ED) Administered 12, 18 or 24 Hours Prior to Intercourse	Drug: Levitra (Vardenafil, BAY38-9456) Drug: Placebo	
25.	The Therapy of Nebido as Mono or in Combination With PDE-5 Inhibitors in Hypogonadal Patients With Erectile Dysfunction	Drug: Testosterone Undecanoate,1000 mg	Siriraj Hospital Bangkoknoi, Ramathibodi Hospital Rajthevee, King Chulalongkorn Memorial Hospital Bangkok, Thailand
26.	A Randomised Study of Levitra to Treat Men With Erections Problems and Previously Untreated With Similar Therapy.	Drug: Levitra (Vardenafil, BAY38-9456) Drug: Placebo	Bruxelles-Brussel, Belgium Drieslinter, Belgium Genk, Belgium
27.	A Safety and Efficacy Study of Prograf in the Prevention of Erectile Dysfunction After Radical Prostatectomy	Drug: Tacrolimus Drug: Placebo	Ann Arbor, Michigan, United States New York, New York, United States
28.	Study of Vardenafil in Patients Suffering From Erectile Dysfunction and Metabolic Syndrome	Drug: Vardenafil (Levitra, BAY38-9456) Drug: Placebo	Cham, Bayern, Germany Regensburg, Bayern, Germany Frankfurt, Hessen, Germany
29.	Exploratory CSII Trial on Erectile Dysfunction in T2DM Patients	Drug: Insulin	Jothydev’s Diabetes & Research Center Thiruvananthapuram, Kerala, India
30.	Study Evaluating the Effects of Avanafil on Semen Parameters	Drug: avanafil Drug: Placebo	Research Facility Huntsville, Alabama, LA, California, San Diego, California, United States
31.	Correlation Study of Vascular Parameters in Hypertensive Men With Erectile Dysfunction	Drug: Vardenafil	Hospital Universitário Pedro Ernesto Rio de Janeiro, Brazil
32.	A Study of Tadalafil After Radical Prostatectomy	Drug: Tadalafil Drug: Placebo	Kortrijk, Belgium Leuven, Belgium Liège, Belgium
33.	Assessment of Duration of Erection With Vardenafil 10 mg	Drug: Levitra (Vardenafil, BAY38-9456) Drug: Placebo	
34.	Levitra^®^—Real Life Safety and Efficacy of Levitra	Drug: Levitra (Vardenafil, BAY38-9456)	
35.	Early Intervention for Erectile Dysfunction After Laparoscopic Resection for Rectal Cancer	Other: vacuum erection device	Department of General Surgery, Nanfang Hospital of Southern Medical University Guangzhou, Guangdong, China
36.	A Study of Semen Characteristics After 9 Months of Daily Tadalafil 20 mg	Drug: tadalafil Drug: placebo	Bothell, Washington, United States
37.	Bioequivalence Study Comparing Two Test Products With One Reference Product, All Containing 5 mg Yohimbine	Drug: Yohimbine	SocraTec R&D GmbH Erfurt, Germany
38.	Assess Efficacy in Subjects With Traumatic Spinal Cord Injury	Drug: Vardenafil (Levitra, BAY 38-9456), 10 mg Drug: Placebo Drug: Vardenafil (Levitra, BAY 38-9456), 20 mg	Badalona, Barcelona, Spain Toledo, Spain

**Table 2:** Overview of manuscripts describing the application of lipid-based systems in encapsulating ED drugs.

S. No	ED Drug	Lipid Based System	Route/Mode of Administration	Ref.
1.	Avanafil	Lipsome	Transdermal	[38]
		Nanoethosome	Transdermal	[39]
		Solid lipid nanoparticle	Transdermal	[40]
		Self-emulsifying drug delivery systems	Oral	[41]
		Nano complex	Oral	[2]
2.	Vardenafil	Nanoethosome	Transdermal	[42]
		Self-emulsifying drug delivery systems	Oral	[43]
3.	Sildenafil Citrate	Nanotransfersome	Transdermal	[44]
		Solid lipid nanoparticle	Oral	[45]
4.	Papaverine Hydrochloride	Nanotransfersome	Transdermal	[46]
5.	Tadalafil	Nano lipid carrier	Transdermal	[47]
6.	Tadalafil and alpha lipolic acid	Self emulsifying drug delivery systems	Transdermal	[48]

**Table 3:** List of granted patents on ED from 2016 to 2021.

Jurisdi-ction	Publication Year	Application Number	Title	Applicants
US	2021	US 16355248	Erectile dysfunction treatment system and method	Sergio Castaneda
US	2021	US 201916597212 A	Composition for treating erectile dysfunction by orchisanatolica extract	Jordan Univ Of Science And Technology
US	2021	US 201916444906 A	Compositions and methods for treating, inhibiting the onset, and slowing the progression of erectile dysfunction including naturally occurring age related erectile dysfunction	K L R M Llc
US	2021	US 201916266293 A	Method for treating organic erectile dysfunction	Bmr Medical Llc
US	2021	US 201715793905 A	Implantable electroacupuncture device and method for treating erectile dysfunction	Valencia Tech Corporation;;Valencia Bioscience Inc
RU	2021	RU 2020111687 A	Method of treating erectile dysfunction in patients suffering radical prostatectomy or brachytherapy	FederalnoeGosudarstvennoeByudzhetnoeUchrezhdenieNatsionalnyjMeditsinskijIssledovatelskijTsent
US	2021	US 202117214142 A	Device for managing male urinary incontinence and reducing erectile dysfunction	Mohamed Adel W
US	2021	US 201815979265 A	Compositions and methods useful in treatment of lower urinary tract symptoms, benign prostatic hyperplasia, erectile dysfunction and other diseases or symptoms	NaturexInc
US	2021	US 201916532367 A	Compositions and methods useful in treatment of lower urinary tract symptoms, benign prostatic hyperplasia, erectile dysfunction and other diseases or symptoms	NaturexInc
RU	2020	RU 2019128290 A	Agent for treating erectile dysfunction	Pivipi Labs Pte Ltd.
US	2020	US 201715710914 A	Methods for the treatment of erectile dysfunction by human birth tissue material composition	BiodlogicsLlc
US	2020	US 201715434226 A	Compositions and methods for treatment of erectile dysfunction	Caprio James J
US	2020	US 201815878423 A	Methods and devices for treating erectile dysfunction	Ohh Med Medical Ltd.
RU	2020	RU 2020107786 A	Method of treating patients with erectile dysfunction	FederalnoeGosudarstvennoeByudzhetnoeUchrezhdenieNatsionalnyjMeditsinskijIssledovatelskijTsent
US	2020	US 201815873000 A	Method of implanting a penile prosthetic in treating erectile dysfunction	Coloplast As
RU	2020	RU 2019125324 A	Portable device for personal diagnosis of erectile dysfunction symptoms	Andreev YurijGermanovich
US	2020	US 201715652258 A	Topical medication method for erectile dysfunction	Lange Iv Carl W
US	2019	US 201615133840 A	Treating erectile dysfunction by orchisanatolica extract	Jordan Univ Of Science And Technology
RU	2019	RU 2018133729 A	Method of treating premature ejaculation and erectile dysfunction	FederalnoeGosudarstvennoeByudzhetnoeUchrezhdenieNovosibirskijNauchnoIssledovatelskijInstitut
RU	2019	RU 2019106747 A	Method of treating erectile dysfunction	FederalnoeGosudarstvennoeByudzhetnoeUchrezhdenieNatsionalnyjMeditsinskijIssledovatelskijTsent
US	2019	US 201615064162 A	Method for treating organic erectile dysfunction	Bmr Medical Llc
RU	2019	RU 2018127153 A	Method of treatment of peyronie’s disease, complicated by erectile dysfunction	FederalnoeGosudarstvennoeByudzhetnoeUchrezhdenieNovosibirskijNauchnoIssledovatelskijInstitut
US	2018	US 201715618751 A	Methods and devices for treating erectile dysfunction	Ohh Med Medical Ltd.
US	2018	US 201815982713 A	Methods of treating erectile dysfunction	BiomimetixJvLlc;;Nat Jewish Health
KR	2018	KR 20170084904 A	Composition for preventing improving or treating erectile dysfunction comprising cavernous derived exosome	UnivInha Res & Business Found;;PostechAcadInd Found
RU	2018	RU 2016134676 A	Method for treatment of chronic abacterial prostatitis/chronic pelvic pain syndrome, combined with erectile dysfunction	ChurakovAleksejArkadevich
US	2017	US 201615085304 A	Method for treating erectile dysfunction	Hussein Hany
KR	2017	KR 20160177086 A	Medical device for erectile dysfunction	Ideafactoryofchoebyongchul Corp
RU	2017	RU 2016127046 A	Method for treatment of patients with erectile dysfunction	FederalnoeGosudarstvennoeByudzhetnoeObrazovatelnoeUchrezhdenieVysshegoObrazovaniyaSamarskij G
RU	2017	RU 2016121765 A	Method for erectile dysfunction treatment	Fed GosudarstvennoeAvtonomnoeObrazovatelnoeUchrezhdenieVysshegoObrazovaniyaPervyjMoskovskij G
